# Clinical genome sequencing in patients with hereditary breast and ovarian cancer: Concept, implementation and benefits

**DOI:** 10.1016/j.breast.2025.104505

**Published:** 2025-05-15

**Authors:** Dennis Witt, Marc Sturm, Antje Stäbler, Benita Menden, Lisa Ruisinger, Kristin Bosse, Ines Gruber, Andreas Hartkopf, Silja Gauß, German Demidov, Nicolas Casadei, Elena Buena Atienza, Kira Mehnert, Janna Witt, Caspar Gross, Leon Schütz, Christopher Schroeder, Stephan Ossowski, Andreas Dufke, Tobias B. Haack, Olaf Riess, Ulrike Faust

**Affiliations:** a Institute of Medical Genetics and Applied Genomics, University of Tübingen, Tübingen, Germany; b Universitäts-Frauenklinik, University of Tübingen, Tübingen, Germany; c NGS Competence Centre Tübingen (NCCT), University of Tübingen, Tübingen, Germany; d Klinikum Sindelfingen-Böblingen Kliniken Böblingen, Böblingen, Germany

**Keywords:** Hereditary breast and ovarian cancer, HBOC, Clinical genome sequencing, Short read sequencing, Polygenic risk scores, PRS, Next-generation sequencing

## Abstract

Hereditary breast and ovarian cancer (HBOC) is one of the most frequent genetic cancer predisposition syndromes. Individuals at risk are identified mainly by family history and histopathological criteria. The current standard genetic testing is exome or panel sequencing. However, many high-risk families remain genetically unexplained. Genome sequencing has the potential to increase the diagnostic yield. This single-center real-world study aims to evaluate advantages of short-read genome sequencing (GS) in HBOC families. We report genome sequencing results of 818 index patients, who fulfilled clinical criteria for genetic testing. Data analysis showed less sequencing gaps and a more uniform coverage compared to a large cohort of in-house exomes. Samples were sequenced at an average depth of 41.2x for the HBOC core genes. Pathogenic variants were found in 9 of 13 core genes in 12.2 % of the patients. GS allowed the classification of a *BRCA1* duplication and detected a whole-exon inversion in *BARD1,* as well as a deep intronic *CHEK2* variant. Furthermore, we successfully used the BRIDGES-PRS in our HBOC cohort and found a significant effect size compared to the control cohort (p = 4.804^−14^, Cohen's-D: 0.476), proving the transferability to a German cohort. GS offers a wealth of information, including the improved detection of structural variants, copy number variants, and parallel detection of complex genetic markers. This has the potential for future analyses, including intronic and intergenic regions. Finally, it also allows for a more streamlined process by converging several tests into one. The approach presented will give guidance for the implementation of GS in HBOC diagnostics.

## Highlights

• WGS in routine HBOC diagnostics offers key insights for future applications and research.
• Transcriptome data enhance HBOC diagnostics and provide novel, valuable insights.
• Large German HBOC cohort (800+ patients) independently validates BRIDGES PRS using genetic data.

## 1 Introduction

Hereditary breast and ovarian cancer syndrome (HBOC) is associated with an increased risk of developing different types of cancer. It commonly results in an earlier onset of cancer in affected patients compared to the general population. To date, several hundred pathogenic variants in more than a dozen genes have been associated with HBOC. *BRCA1* and *BRCA2* were the first genes identified to be associated with HBOC, and together with *TP53* and *PALB2*, are considered high-risk genes for breast cancer. Causative variants in *BRCA1* and *BRCA2* account for a majority of up to 80 % of all genetically confirmed HBOC diagnoses [1,2]. Due to the increased cancer risk, surveillance programs and prophylactic surgery are offered to carriers of pathogenic variants in HBOC-genes, depending on the individual risk profile [3]. Previous studies have demonstrated the effectiveness of these measures and their relevance for therapy [4]. The predisposition often affects multiple family members.

Today, a relevant part of cases fulfilling clinical criteria for HBOC remain genetically unexplained [5,6]. Even with a focus on extended families, no major risk gene with high life-time risks has been discovered in the last decade. Although additional genes associated with HBOC are indeed under discussion, the German Consortium for Familial Breast and Ovarian Cancer currently classifies these as research genes, which are not yet incorporated into standard diagnostic procedures. An example of such a gene is *MAP3K1*, whose impact is being evaluated as potentially comparable to that of *PALB2* [7].

However, reports on Genome Sequencing (GS) integration for other disease groups raise hopes that short-read GS might identify relevant genetic alterations in intronic or regulatory regions of known and yet unknown cancer genes in the future [[8], [9], [10], [11], [12], [13], [14]]. Although GS can detect structural and intronic variants [15], the functional impact of selected variants was thought to be difficult to predict from the start of implementation. Therefore, we decided on implementing a second readout, in the form of blood transcriptome sequencing (TS), to further increase the diagnostic yield [16]. For HBOC cases we have also implemented a genome based polygenic risk score (PRS)-examination, based upon the BRIDGES-PRS [17]. The BRIDGES PRS is an adaptation of the Mavadatt PRS, which was developed on SNP array data. The BRIDGES-PRS consists of 306 SNPs, of which 295 are identical to the Mavaddat 313 PRS. It was optimized to eliminate surrogate markers and to better align with NGS results [17,18].

This was decided to gain additional knowledge, as HBOC cases remain often unsolved or unclear. PRS have significantly increased their accuracy over the last years, which may explain additional HBOC-cases [19] Recent publications report on the utility of combining PRS information with other known risk factors to more accurately predict individual risk estimates. A widely used calculator is the CanRisk webtool (https://www.canrisk.org/). However, due to the fact that they include a large number of polymorphisms, it is unlikely that the PRS will explain familial cases of breast cancer with seemingly dominant inheritance pattern [[20], [21], [22], [23], [24]].

An additional topic of our study are findings in so called actionable genes, as GS facilitates the detection of pathogenic variants in genes, which are not included in the HBOC-panel. Pathogenic variants in actionable genes are typically linked to treatable and/or preventable diseases. Former studies of our research group presented an average rate of 5 % of patients harboring pathogenic/likely pathogenic variants within an ACMG59 based gene list [25]. The specific list of genes was developed by the German Network on Actionable genes.

As former publications of our group reported, we implemented GS at our institute for a broad range of diseases in clinical care [25] and started to offer GS to HBOC families in 2022. A large cohort of patients with rare diseases and HBOC families were sequenced since. The study at hand was conceptualized to highlight advantages and challenges of GS in clinical care and therefore provides guidance for implementation of GS.

## 2 Materials and methods

### 2.1 Patient cohort and target genes

All patients were recruited from our outpatient clinic for familial cancer predisposition. Included patients were affected by at least one disease, breast cancer or ovarian cancer, and concurrently met the diagnostic HBOC criteria [26]. The criteria may also be found in the supplement tables. Thirteen genes were defined as the scope of this examination: *BRCA1, BRCA2, PALB2, RAD51C, RAD51D, BRIP1, BARD1, PTEN, CDH1, STK11, TP53, CHEK2*, and *ATM*. Additionally, patients were asked to consent to reporting of actionable genes, as suggested by the ACMG and German Network for Actionable Genes (GNAG). A control cohort, needed for PRS calculation, was recruited from an in-house cohort of patients and consists of 1000 individuals. The inclusion criteria were defined as being adult and not suffering from any type of cancer. This study was approved by the local IRB (project number 133/2021BO1). Patients with additional TS provided consent to participate in the Ge-Med project study (NCT04760522). Recruitment took place between April 2019 and March 2023.

During the course of the study, 19 patients, taken from a different cohort, were sequenced with long read (lr)-genomes, the corresponding quality metrics will also be discussed in this study. However, these patients were anonymized, and their disease and characteristics are not disclosed in this paper.

### 2.2 Exome and short-read genome sequencing

The lab is diagnostically accredited for exome sequencing (ES) and GS (DAkkS DIN EN ISO 15189). ES and GS, as well as subsequent variant filtering were performed as previously described [14,27]. In brief, coding genomic regions were enriched for ES from whole blood genomic DNA, using a SureSelect XT Human All Exon Kit V.6 or V.7 (Agilent Technologies). Prepared libraries were sequenced as 2x125bp or 2x100bp paired-end reads on a NovaSeq6000 system (Illumina). For GS, genomic DNA was extracted from whole blood, using FlexiGene DNA kits (Qiagen) and further processed using the TruSeq PCR-Free Library Prep kit (Illumina). Libraries were sequenced as 2x152bp reads on a NovaSeq6000 system to an average depth of more than 30x. Downstream data processing, ethnicity calculation and variant interpretation was done using the megSAP pipeline (https://github.com/imgag/megSAP) enabling the detection of different types of genomic variation including single nucleotide variants, small insertions and deletions, repeat expansions, and structural variants (copy number variants, inversions, translocations). Ancestry was calculated by small nucleotide polymorphism (SNP) clustering. Prioritized sequence variants were classified according to the guidelines of the American College of Medical Genetics and Genomics. All structural variants mentioned in this paper were validated with multiplex ligation-dependent probe amplification (MLPA) (MLPA Kit P002D1, P045D1, P489A1, P041B1, P042B2, P190D1; MRC Holland). Notations are given in GRCh38.

### 2.3 Long-read genome sequencing

The lab is also diagnostically accredited for lrGS (DAkkS DIN EN ISO 15189). Genomic integrity was assessed using pulse-field capillary electrophoresis with the Genomic DNA 165 kb Analysis Kit on a FemtoPulse (Agilent) instrument. Quantitation of DNA was assessed using the dsDNA High Sensitivity assay on a Qubit 3 fluorometer (Thermo Fisher) and purity was assessed by Nanodrop. A total of 3 μg of genomic DNA was sheared with Megaruptor 3 (Diagenode). Library was prepared with the 1D Ligation SQK-LSK109-XL Sequencing kit (Oxford Nanopore Technologies). Library average fragment length was assessed using the Genomic DNA 165 kb Analysis Kit on a FemtoPulse (Agilent). A total of 400–600 ng (30–50 fmol) of library was loaded on PromethION R9.4.1 flow cells. Downstream data processing and variant interpretation was done using the megSAP pipeline (https://github.com/imgag/megSAP).

Short-read Transcriptome Sequencing.

Blood samples were collected using PAXgene blood tubes. RNA isolation was performed using the QIAsymphony PAXgene Blood RNA kit on the QIAsymphony SP platform using the protocol PAXgen RNA V5 (Qiagen). Post isolation, the RNA was eluted in RNase-free water. RNA concentration was measured using the Qubit Fluorometric Quantitation and RNA Broad-Range Assay (Thermo Fisher Scientific). RNA Integrity Number (RIN) was determined using the Fragment Analyzer 5300 and the Fragment Analyzer RNA kit (Agilent). For library preparation, the mRNA fraction was enriched using poly(A) capture from 100 ng of total RNA using the NEBNext Poly(A) mRNA Magnetic Isolation Module (New England Biolabs). Subsequently, libraries were prepared using the NEBNext Ultra II Directional RNA Library Prep Kit for Illumina (New England Biolabs). Library preparations were performed using the liquid handler Biomek i7 (Beckman). Library molarity was determined by measuring the library size using the Fragment Analyzer 5300 and the Fragment Analyzer DNA HS NGS fragment kit (Agilent) and the library concentration (>5 ng/μl) using Qubit Fluorometric Quantitation and dsDNA High sensitivity assay (Thermo Fisher Scientific). The libraries were denatured according to the manufacturer's instructions, diluted to 270 pM and sequenced as paired-end 100 bp reads on an Illumina NovaSeq 6000 (Illumina). The TS aimed to achieve a depth of approximately 50 million clusters per sample. Downstream data processing and variant interpretation was done using the megSAP pipeline (https://github.com/imgag/megSAP).

### 2.4 Risk calculations

To establish a baseline for comparing GS results, we have initially calculated the probabilities for finding pathogenic germline variants by processing the patients’ family history with the CanRisk webtool (CanRisk version: v2.4.1 (2024-04-05)). Where available, we included information about clinical parameters such as breast tissue density, menarche, climacteric, endometriosis, tubal ligation, surgical removal of ovarian or breast tissue, and lifestyle information.

CanRisk was also used to model lifetime risks for the development of breast cancer by adding PRS-results. For details on the methods of PRS calculation, see the following section.

### 2.5 Polygenic risk scores (PRS)

Polygenic risk score for SNP-based breast cancer, was calculated as suggested by Mavaddat [17,19]. For this, all SNPs had to be lifted to GRCh38. The lifting was done via the Lift Genome Annotations Tool from UCSC.

The calculation's initial outputs were raw-PRS values, z-scores were calculated with the above mentioned in-house cohort of 1000 adult individuals suffering from diseases other than cancer.

### 2.6 Statistical analysis

Statistical data analysis was performed using Python v.3.6. Where applicable, t-tests were performed. For this SciPy v.1.14.0 was used. When the prerequisites for t-tests were not fulfilled due to non-Gaussian-feature distributions, we used a Man-Whitney-U-Test. Figures were created with Matplotlib v.3.7.1 and Seaborn v.0.12.2. For comparing expected germline findings against the actual observed findings, a binomial approach was employed.

## 3 Results

Genome sequencing was implemented at our institute in 2022 for the majority of genetic tests, which are performed for rare diseases as well as cancer predisposition. Over the last two years, we collected a large dataset of 7580 patients with different genetic conditions and more than 800 patients with suspected hereditary breast and ovarian cancer syndrome.

### 3.1 Genome sequencing in a large cohort of HBOC patients

Our HBOC cohort. Over the course of two years, we analyzed 815 patients with either ovarian cancer (n = 106), breast cancer (n = 696), or both (n = 13). The complete cohort consisted of 807 women and 8 men. A total of 778 patients were of European ancestry and 661 were diagnosed with isolated breast cancer. The average age at onset was 50.11 years. 104 European patients were diagnosed with isolated ovarian cancer with an average age at onset of 57.67 years. All patients with breast and ovarian cancer were of European descent. Their mean age at the first diagnosis was 56.77 years. Detailed information can be found in the supplement.

Quality characteristic of GS. On average more than 916 million reads were generated per patient and an average of 129,492.29 high-quality variants were called within the coding regions. Of all variants, 99.25 % were known polymorphisms. An average sequencing depth of 41.16x was achieved across the exons of the 13 HBOC genes. In comparison to a large in-house cohort of exome data we observed a lower average overall sequencing depth (144.96x vs 41.16x), while GS had a superior target region 30x coverage (96.32 % vs 93.56 %; p = 1.601x10-80). Also, GS showed a lower number of sequencing gaps (defined as a sequencing depth of less than 20 reads) for seven moderate risk genes (see Table 1). Sequencing gaps remained comparable for the other genes and were in total <0.5 %. It should be mentioned that even if a gap was detected in GS bioinformatically (i.e. less than 20 reads), a sufficient number of reads was available in most cases to complete diagnostic analysis by visual inspection (between 15 and 20 reads). Lower read counts would have been subjected to further sequencing.

**Table 1:** **Comparison of srGS and ES in the Identification of gaps.** Shown are the different HBOC-genes analyzed and gaps presented as percentage of the gene.

Gene	SrGS(%)	ES(%)	p-value
*BRCA1*	0.09	0.001	3.81x10^-21
*BRCA2*	0.02	0.13	0.048
*BRIP1*	0.11	0.09	1.45x10^-27
*PTEN*	0.31	0.04	3.14x10^-11
*TP53*	0.03	0	0.0004
*PALB2*	0.21	0.33	1.78x10^-80
*RAD51C*	0.01	0.03	0.0003
*RAD51D*	0.003	0.01	7.77x10^-5
*BARD1*	0.14	0.50	2.28x10^-240
*CDH1*	0.01	0.04	2.59x10^-23
*CHEK2*	0.04	0.2	6.24x10^-37
*ATM*	0.07	0.61	2.98x10^-210
*STK11*	0.0004	0.0020	0.65

Genetic Findings. Thirteen HBOC-related genes were analyzed and 100 likely pathogenic or pathogenic (LP/P) germline variants were found in 97 patients. This equals a detection rate of 12.2 %. The majority of variants were found, as expected, in *BRCA1* (35), *BRCA2* (22), *CHEK2* (18), and *ATM* (12). Interestingly, one patient carried two LP/P variants (*BRCA2* & *CHEK2*). The LP/P variants encompass 62 Loss-of-function (LoF)-variants, 19 Missense-variants, one synonymous variant, nine splice-variants and nine structural aberrations, which (likely) affect protein function. Additionally, a total of 131 variants of uncertain clinical significance (VUS) were identified. CanRisk calculations, which incorporated clinical and familial information, yielded a mean expected finding rate of 13 %. When compared with the actual results, no significant deviation was observed (p-value: 0.3, z-value: 1.0364). The availability and scope of the clinical information varied. Of the 696 breast cancer patients, 628 provided self-assessment information by a questionnaire regarding clinical details.

**Fig. 1:**
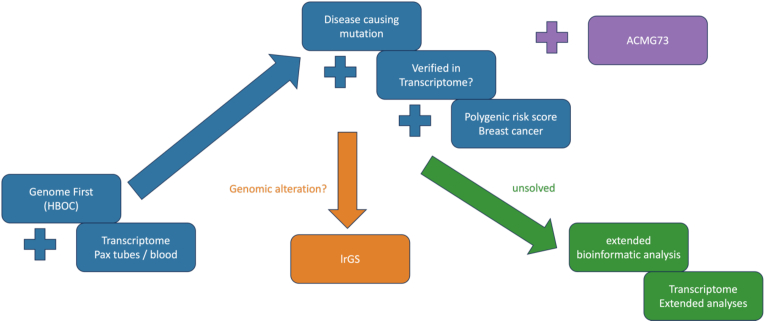
Study flow chart: HBOC diagnostic concept. Blue: routine diagnostics, which were performed for every patient; orange: case based verification of insertion site of duplicated regions of unsolved, structural variants; green: case based extended research; pink: optional screening for (likely) pathogenic variants in actionable genes. (For interpretation of the references to colour in this figure legend, the reader is referred to the Web version of this article.)

Polygenic risk scores. In this study, the BRIDGES-PRS was calculated for all patients. Further analysis was conducted for 661 breast cancer patients of European heritage. Overall, 300 of 306 BRIDGES-SNPs were found in our cohort, absent were the following SNPs: GrCH38: chr1:117598870 C > A rs12406858, chr2:69945455 A > G rs6756513, chr5:82217128 T > TA rs146817970, chr11:1874478 A > G rs4980386, chr20:54680310 A > G, chr20:11399194 C > T rs1154723. For all 815 patients, GS and subsequent BRIDGES-PRS-calculation were performed. However, only the PRS-results of European patients with breast cancer (n = 661) were further analyzed. PRS reports were integrated into the CanRisk risk assessment. The diagnostic workflow is shown in Fig. 1. For the control and HBOC-cohort, a normal distribution of z-scores was found. The PRS values (z-score) of our combined breast cancer cohort was significantly higher than in our local controls (p = 3.906∗10–14, Cohen's-D: 0.382, r = 0.188) (Supplementary Fig. 1). Furthermore, the z-score was significantly higher for patients without a reported LP/P variant in the core genes (n = 498) than in patients with a known causative variant (n = 81) (0.42 versus 0.19, p-value: 5.022∗10-2) (Supplementary Fig. 2). In total, 130 of the breast cancer patients (20.8 %) were attributed to the 90th percentile or higher, thirteen of these had a significant finding in the HBOC-genes. The mean age of disease onset for this group was found to be 50.16 years, which mirrors the average age of the examined cohort.

Secondary Findings. A total of 663 patients also consented to the reporting of actionable genes. Overall, 23 additional LP/P variants with medical relevance were identified. These were found in 21 patients resulting in a rate of 3.02 % (20/663) of patients with a secondary finding. Secondary findings were found in *HFE, MUTYH, MYBPC3, LDLR, GLA, APOB, KCNH2, ATP7B, TTN, TSC1, MLH1, and SDHB*. All five patients, who had findings in cancer predisposition genes (*MLH1* & *MUTYH*), did not report familial cancer cases, typically associated with findings in these genes. Additionally, they were not affected by an associated cancer themselves.

### 3.2 Selected cases illustrating the strengths of GS

**Fig. 2:**
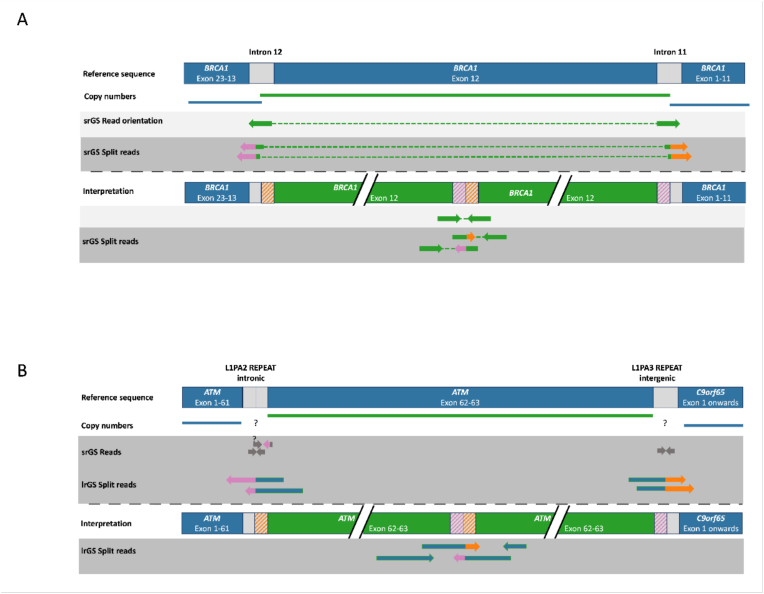
Structural variants of interest (visual illustration): Structural variants detected by short-read GS and long-read GS. Panel A shows a tandem duplication of exon 12 of *BRCA1* identified by short-read GS. The detection of a tandem duplication is possible through the interpretation of split reads from which information regarding the localization of the breakpoints can be obtained. Panel B presents a tandem duplication of exons 62 and 63 of the *ATM* gene, identified by long-read GS. Short-read GS did not span the flanking repeats and therefore the exact breakpoint position could not be detected. Question marks in panel B indicate the potential location of the break point and uncertainty on a base pair resolution due to the repetitive structure.

GS has advantages over conventional genetic tests detecting structural and intronic/regulatory variants. We were able to identify a deep intronic, likely pathogenic *CHEK2*-variant in one patient (NM_007194.4:, c.1009-118_1009-87delinsC). We were able to describe the breakpoints of CNVs on basepair level in three additional patients, supporting variant classification and characterization of variants. In addition, we found three structural variants in our overall cohort of patients with GS. This included a pathogenic inversion of *BARD1* with intronic breakpoints, a likely pathogenic partial duplication of exon 13 in *BRCA1*, and a likely pathogenic tandem duplication in *ATM*. The duplication in *BRCA1* was found to be a tandem duplication that disrupts the reading frame of *BRCA1* which would not have been seen by conventional diagnostics and therefore the variant would not have been classified properly (panel A Fig. 2). The *BARD1* inversion would not have been identified by conventional diagnostics because of missing copy-number changes.

### 3.3 New protocols and complementary sequencing approaches

Long-read genome sequencing. We compared our short read GS results to long-read GS and evaluated if sequencing gaps could be further reduced with long-read GS and if long-read GS offers additional value compared to short-read sequencing. We compared the results to a cohort of 19 in-house long-read GS samples (non-HBOC patients), sequenced with ONT chemistry and R9.4.1 pores. An average depth of 36.5x (range of 26.23–48.20) was achieved and we observed a correlation of mean sequencing depth with the fraction of gaps. Sequencing gaps were absent starting at a depth of 38x in long-read GS. Another interesting aspect of long-read GS is its superior capacity to characterize structural variants. This is shown by the identification of precise break points of the above described *ATM* tandem duplication of exons 62 and 63 which were located in repeats and therefore not identifiable by other methods (panel B Fig. 2).

RNA analysis was performed for a subset of 127 HBOC patients. TS was used to see if additional information on detected variants could be generated and if variant classification could be improved. The average read count was 121 million reads with an average fraction of house-keeping genes of 16.55 %. The expression levels for most of the HBOC genes were low or near zero, which is especially important for *BRCA2*. Further information regarding the RNA-expression can be found in Supplementary Fig. 3. Due to low expression levels, results should be interpreted with caution. Nevertheless, it was possible to confirm 60.87 % of LP/P germline variants on the RNA level (14/23). We confirmed 3/3 *CHEK2*, 3/3 ATM, 0/2 *BARD1*, 1/1 *RAD15C*, 0/4 *BRCA2*, and 7/10 *BRCA1* variants. Interestingly, while all of the variants in *CHEK2* and ATM were confirmed, we could not verify any of the *BRCA2* variants most likely due to its low expression in peripheral blood.

## 4 Discussion

The current diagnostic standard for HBOC testing involves targeted panel sequencing or ES, with detection rates below 25 %, depending on the patient selection criteria and tested genes [5,6]. However, these methods have limitations related to the detection of structural variants, deep intronic variants or variants in regulatory regions [14]. GS has demonstrated superior detection rates in other disease fields [[8], [9], [10], [11], [12], [13], [14]], but comprehensive data in larger HBOC cohorts are lacking.

In this study, the overall detection rate of LP/P variants in 13 HBOC genes was 12.2 %. While some studies report similar detection rates, it is lower compared to previous studies in a similar cohort [5,28]. Our results are supported by the calculated detection rate of CanRisk. Several possible explanations for the differences were considered: Firstly, there may be a cohort bias. The local cohort presented is composed, to a significant extent, of elderly women with extended pedigrees. When considering the background prevalence of breast cancer of approximately 12 %, this may lead to a bias regarding the first of the consortial criteria. An additional explanation may be attributed to a re-testing bias: While efforts were made to select patients for initial examination, the possibility that some patients had previously undergone negative genetic testing with panel or exome sequencing cannot be excluded. However, considering the CanRisk predictions, which demonstrate high accuracy, it is more likely that (some of) the criteria used for patient selection failed to enrich the patient cohort with mutation carriers to the intended degree.

Genome sequencing was able to detect complex structural variants in *BRCA1*, *BARD1*, and *ATM*, which would not have been accurately detected or classified using current standard genetic tests. Due to the intronic coverage of GS, we were able to identify retrospectively a *CHEK2* variant in a patient, allowing us to solve this case purely by data reanalysis. The mentioned deep intronic variant was published during the course of this study [29]. Scientifically valuable data were collected in both intergenic and intronic areas. For several HBOC-genes, some intronic variants were already reported: Such as *BRCA1* [30], *BRCA2* [31], *ATM* [32] and *CHEK2* [29]. Process-wise GS has additional benefits by simplifying our diagnostic pipeline, enabling not only our technicians but also our geneticists to perform genetic diagnostics with a one-fits-all approach, reducing the necessary amount of discussion and coordination between departments.

Another benefit of GS lies in the genome-wide detection of SNPs, which allows the calculation of different PRSs. The study demonstrated the efficacy of the BRIDGES-PRS by calculating its ability to stratify patients with breast cancer from those without the condition. Additionally, a similar effect was detected for informative and non-informative tested patients. However, given that PRSs are based on an association-based rationale, there are significant uncertainties, e.g. regarding their interpopulation transferability [17,[33], [34], [35], [36]]. Identifying the ‘true’ functional variants behind the SNPs and addressing this association bias will be crucial for future applications.

GS provided information about actionable genes and secondary findings. Secondary findings were observed in approximately 3 % of cases across a diverse range of genes associated with various diseases. This observation necessitates contextualization in comparison to the 4.1 % reported in the UK Biobank [37]. The lower incidence rate in our study can be attributed partly to cohort bias and partly to the inclusion of *BRCA1*, *BRCA2*, and *PALB2* genes in the ACMG 73 list, which were also included in this study's panel. Consequently, secondary findings regarding these genes were not possible in our study, thereby reducing the potential incidence rate. We would like to point out that, during the course of this study, a discussion regarding the ethics and relevance of reporting secondary findings emerged among the German genetic scientific community [38]. Among other considerations, the debate focuses on the relevance of missense variants in cancer genes found in unaffected families and issues related to patient consent.

Summarizing our experience, we can clearly state that GS allows for additional use cases and less complex diagnostic pipelines without sacrificing quality. GS rather outperforms our prior ES pipeline regarding a more uniform coverage and reduced number of sequencing gaps in moderate-risk genes. Its sensitivity for structural variants, which are known to cause HBOC [39], makes complex complementary MLPA tests unnecessary, which are needed for enrichment-based approaches like ES [40]. GS allows a higher level of automatization and shorter processing time compared to ES, omitting the time-consuming enrichment step. Finally, GS offers greater flexibility regarding reanalysis of candidate genes, detection of variants in regulatory regions, and structural variants. In contrast, TS was found not to be quite as successful given the low expression of numerous HBOC-critical genes in blood. Despite this, TS holds complementary information to GS in selected cases as it aids in the evaluation of splice variants and can confirm structural variants [16]. It is therefore prudent to consider the addition of TS on a case-by-case basis, taking into account specific variants and their expression in blood cells. Targeted approaches present a viable alternative in these cases.

Also, if available, TS of other biomaterial like breast-tissue or fibroblasts might be more suitable to increase diagnostic sensitivity. Finally, long-read GS holds the promise of improved diagnostic sensitivity and solving more HBOC cases due to its superior detection of structural variants, added information regarding haplotyping, methylation, repeats, pseudogene coverage and mobile element insertions [[41], [42], [43], [44]].

Long-read GS is currently too expensive and bioinformatically complex for routine diagnostics. Therefore, GS is a highly reliable, sensitive, cost-effective and efficient first tier diagnostic for current HBOC-testing in a clinical setting.

## CRediT authorship contribution statement

**Dennis Witt:** Writing – review & editing, Writing – original draft, Visualization, Software, Resources, Methodology, Investigation, Formal analysis, Data curation. **Marc Sturm:** Writing – review & editing, Software, Data curation, Conceptualization. **Antje Stäbler:** Writing – review & editing, Investigation. **Benita Menden:** Writing – review & editing, Resources. **Lisa Ruisinger:** Writing – review & editing, Resources. **Kristin Bosse:** Writing – review & editing, Resources. **Ines Gruber:** Writing – review & editing, Resources. **Andreas Hartkopf:** Writing – review & editing, Resources. **Silja Gauß:** Writing – review & editing, Investigation. **German Demidov:** Writing – review & editing, Software, Investigation, Data curation. **Nicolas Casadei:** Writing – review & editing, Writing – original draft, Resources, Data curation. **Elena Buena Atienza:** Writing – review & editing, Writing – original draft, Investigation, Data curation. **Kira Mehnert:** Writing – review & editing, Data curation, Conceptualization. **Janna Witt:** Writing – review & editing, Data curation. **Caspar Gross:** Writing – review & editing, Software, Data curation. **Leon Schütz:** Writing – review & editing, Software, Data curation. **Christopher Schroeder:** Writing – review & editing, Writing – original draft, Validation, Resources, Project administration, Methodology, Investigation, Conceptualization. **Stephan Ossowski:** Writing – review & editing, Writing – original draft, Supervision, Software, Resources, Data curation. **Andreas Dufke:** Writing – review & editing, Supervision, Resources. **Tobias B. Haack:** Writing – review & editing, Supervision, Resources, Methodology. **Olaf Riess:** Project administration, Methodology, Investigation, Funding acquisition, Conceptualization. **Ulrike Faust:** Writing – review & editing, Writing – original draft, Investigation.

## Ethics approval and consent to participate

This study was approved by the Ethics Committee at the University of Tübingen with the respective identification number: 133/2021BO1.

## Consent for publication

No individual information is published.

## Data availability

All data used and/or analyzed during the current study are available from the corresponding author on reasonable request.

## Funding

The GeMed study was partially supported by 10.13039/100010905Illumina and the German 10.13039/100009647Ministry of Health via the genomDE initiative.

## Declaration of competing interest

Dennis Witt:I have no competing interest to declare.

Ulrike Faust:Has no competing interest to declare.

Marc Sturm:Has no competing interest to declare.

Antje Stäbler:Has no competing interest to declare.

Benita Menden:Has no competing interest to declare.

Lisa T. Ruisinger:Has no competing interest to declare.

Kristin Bosse:Has no competing interest to declare.

Ines Gruber:Has no competing interest to declare.

Andreas Hartkopf:Has no competing interest to declare.

Silja Gauss:Has no competing interest to declare.

German Demidov:Has received research funding by European Union's Horizon 2020 research and innovation programme under grant agreement number 779257 and has received fees for consulting by Diagenius BV, Amsterdam, Netherlands. Both instances are not associated with the current paper.

Nicolas Casadei:Has received research funding by Deutsche Forschungsgemeinschaft (DFG) under the ID INST 37/1049-1 and received payment for lectures at university of Reutlingen.

Elena Buena Atienza:Has received research funding by Deutsche Forschungsgemeinschaft (DFG) under the ID INST 37/1049-1.

Janna Witt:Has no competing interest to declare.

Caspar Gross:Has no competing interest to declare.

Leon Schütz:Has no competing interest to declare.

Stephan Ossowski:Has received research funding by Illumina Inc and Honoraria from Illumina Inc. For a presentation at GfH conference 2023 as well as support for attending meetings at Illumina and ONT in 2023.

Andreas Dufke:Has no competing interest to declare.

Tobias Haack:Has received research funding by Illumina Inc.

Olaf Riess:Has received research funding by Illumina Inc and German Research Foundation and EU funding and German ministry of Health. Olaf Riess is also Representative of the German Ministry of Health in the European + MG Project (unpaid).

Christopher Schroeder:Has received research funding by Illumina Inc.
