# Supplementary material for: Clinical genome sequencing in patients with hereditary breast and ovarian cancer: Concept, implementation and benefits
Source: Breast. 2025 May 15;82:104505. doi: 10.1016/j.breast.2025.104505 (PMC12150180; doi:10.1016/j.breast.2025.104505)
Supplement: Multimedia component 1 [file mmc1.docx]

**Supplementary Material Description**

This manuscript's supplementary material comprises tables (supplementary_tables.xlsx) that augment the primary manuscript's information with a comprehensive characterization of all included patients in the HBOC cohort and their respective findings. This encompasses both HBOC and actionable gene findings. The information is further expanded upon by the BRIDGES PRS data and the specific clinical criteria that led to the inclusion of each patient, as well as the calculated finding rates per patient (CanRisk).

This supplementary data is integral to the study, as it provides complete transparency regarding the results.
