# Supplementary material for: Clinical genome sequencing in patients with hereditary breast and ovarian cancer: Concept, implementation and benefits
Source: Breast. 2025 May 15;82:104505. doi: 10.1016/j.breast.2025.104505 (PMC12150180; doi:10.1016/j.breast.2025.104505)
Supplement: Multimedia component 3 [file mmc3.docx]

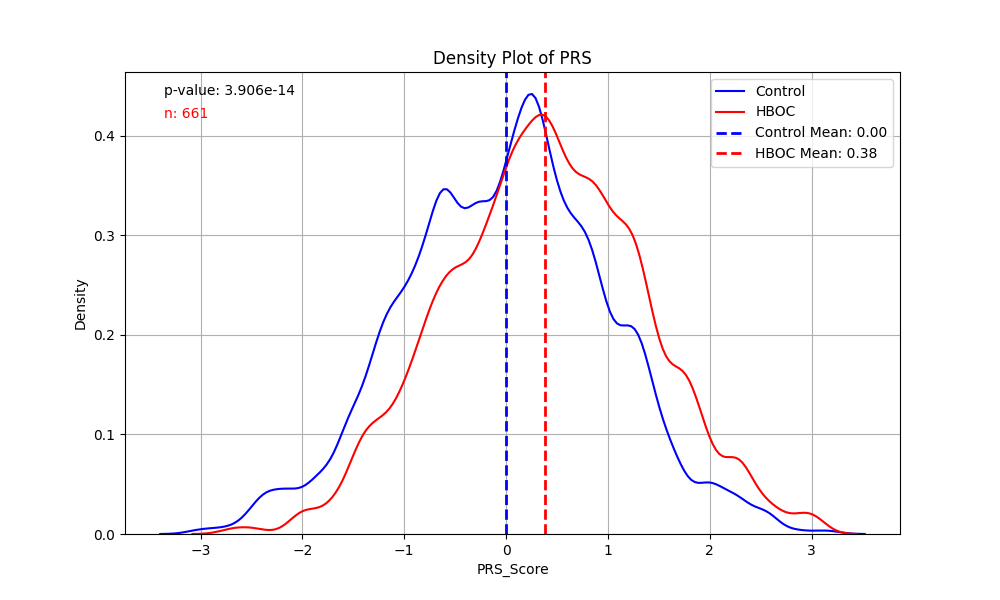
Supplementary figure 1:

Supplementary figure 1 comprises two superimposed density plots depicting the distribution of PRS values for the HBOC (red) and control cohorts (blue). The respective means are indicated by dashed lines.
