# Supplementary material for: Clinical genome sequencing in patients with hereditary breast and ovarian cancer: Concept, implementation and benefits
Source: Breast. 2025 May 15;82:104505. doi: 10.1016/j.breast.2025.104505 (PMC12150180; doi:10.1016/j.breast.2025.104505)
Supplement: Multimedia component 4 [file mmc4.docx]

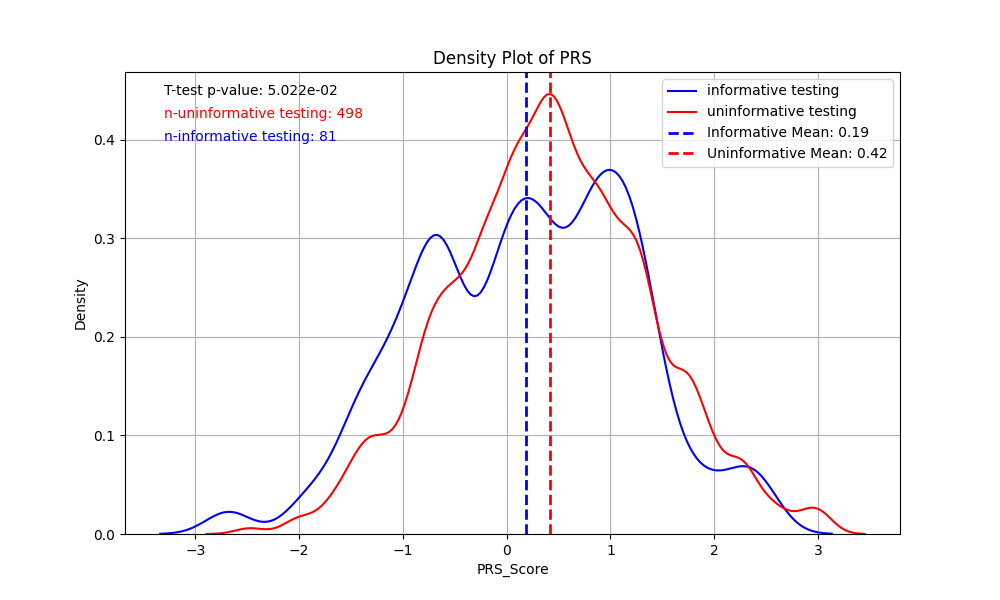
Supplementary figure 2:

Supplementary figure 2 presents two superimposed density plots illustrating the distribution of PRS values for the HBOC cohort, with significant genetic findings (red) and those without (blue). The respective means are indicated by dashed lines.
