# Supplementary material for: Clinical genome sequencing in patients with hereditary breast and ovarian cancer: Concept, implementation and benefits
Source: Breast. 2025 May 15;82:104505. doi: 10.1016/j.breast.2025.104505 (PMC12150180; doi:10.1016/j.breast.2025.104505)
Supplement: Multimedia component 5 [file mmc5.docx]

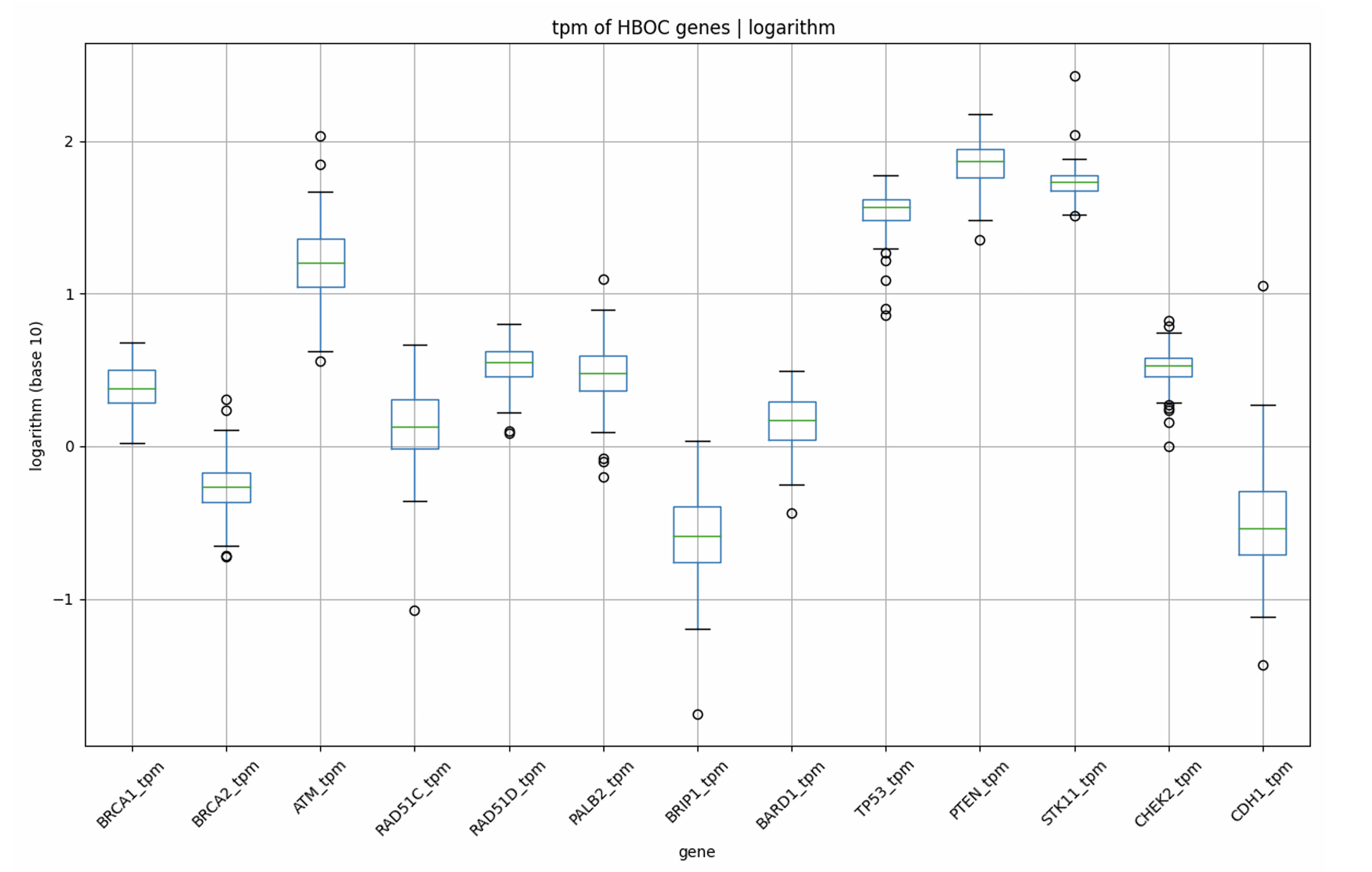


Supplementary figure 3:

Supplementary figure 3 presents a swarm boxplot, which illustrates the RNA-transcript expression values (tpm) in logarithmic fashion for all 13 breast cancer genes that were the focus of this study.
